# Adenosine Receptors in Developing and Adult Mouse Neuromuscular Junctions and Functional Links With Other Metabotropic Receptor Pathways

**DOI:** 10.3389/fphar.2018.00397

**Published:** 2018-04-24

**Authors:** Josep Tomàs, Neus Garcia, Maria A. Lanuza, Manel M. Santafé, Marta Tomàs, Laura Nadal, Erica Hurtado, Anna Simó-Ollé, Víctor Cilleros-Mañé, Laia Just-Borràs

**Affiliations:** Unitat d’Histologia i Neurobiologia, Facultat de Medicina i Ciències de la Salut, Universitat Rovira i Virgili, Reus, Spain

**Keywords:** motor end-plate, postnatal synapse elimination, axonal competition, acetylcholine release, muscarinic acetylcholine receptors, adenosine receptors, neurotrophins, TrkB

## Abstract

In the last few years, we have studied the presence and involvement in synaptogenesis and mature transmitter release of the adenosine autoreceptors (AR) in the mammalian neuromuscular junction (NMJ). Here, we review and bring together the previously published data to emphasize the relevance of these receptors for developmental axonal competition, synaptic loss and mature NMJ functional modulation. However, in addition to AR, activity-dependent mediators originating from any of the three cells that make the synapse (nerve, muscle, and glial cells) cross the extracellular cleft to generate signals in target metabotropic receptors. Thus, the integrated interpretation of the complementary function of all these receptors is needed. We previously studied, in the NMJ, the links of AR with mAChR and the neurotrophin receptor TrkB in the control of synapse elimination and transmitter release. We conclude that AR cooperate with these receptors through synergistic and antagonistic effects in the developmental synapse elimination process. In the adult NMJ, this cooperation is manifested so as that the functional integrity of a given receptor group depends on the other receptors operating normally (i.e., the functional integrity of mAChR depends on AR operating normally). These observations underlie the relevance of AR in the NMJ function.

## Introduction

In addition to the main neurotransmitter-receptor signal, several signaling pathways coordinate the pre- and postsynaptic cells and associated glia in the tripartite synapses in accordance with functional demands. In the NMJ, presynaptic mAChRs directly couple ACh secretion to the regulation of the release mechanism itself (Caulfield and Birdsall, 1998; Slutsky et al., 2001; Minic et al., 2002; Santafé et al., 2003, 2004; Garcia et al., 2005). Moreover, presynaptic nicotinic ACh autoreceptors (nAChRs) are also present at the NMJ (Correia-de-Sá, 1994; Salgado et al., 2000). Also, at the NMJ, the presynaptic neurotrophin and cytokine receptors can be influenced by target-derived signals (Bibel and Barde, 2000; Roux and Barker, 2002; Pitts et al., 2006), and glutamate together with mGluR1 also mediate the signaling in this synapse (Waerhaug and Ottersen, 1993; Lück et al., 2000; Malomouzh et al., 2011; Marmiroli and Cavaletti, 2012; Walder et al., 2013).

Studies in the early 1970s Ribeiro and Walker (1973) showed that adenosine and ATP modulate the presynaptic component through purinergic receptors (adenosine P1Rs and ATP P2Rs) (Correia-de-Sá et al., 1991; Ribeiro et al., 1996). The first authors describing adenosine effects at the mammalian NMJ were (Ginsborg and Hirst, 1971, 1972; see also Singh et al., 1986). It is known that both nerve and muscle cell activity can contribute to the extracellular adenosine release (Cunha and Sebastião, 1993; Ribeiro et al., 1996).

In the last few years, we have studied the presence and involvement of AR in the synaptogenesis and transmitter release in the developing and mature mammalian NMJ (Garcia et al., 2013; Tomàs et al., 2014; Tomàs J. et al., 2017; Santafé et al., 2015; Nadal et al., 2016a,b, 2017). Here, we review and bring together previously published data to contribute to emphasizing the relevance of these receptors in this synapse. Moreover, we also discuss our previous studies in relation with the interaction between AR, mAChR and the TrkB in the control of synapse elimination during development and transmitter release in the adult NMJ.

Our results indicate that during NMJ synaptogenesis, AR (A_1_R and A_2A_R subtypes) contribute to the developmental synapse elimination process, helping to define the winner of the competition between axon terminals. In the adult, AR help to modulate transmitter release by limiting spontaneous quantal leak of ACh and preserve synaptic function by reducing depression during repetitive activity. To realize these functions, several synergistic and antagonistic relations exist between AR and, at least, the mAChR subtypes (M_1_, M_2_, and M_4_) and the TrkB receptor. These observations underlie the relevance of AR in the NMJ function.

## Adenosine Receptors Localization in the Nmj

Four subtypes of AR have been cloned (A_1_R, A_2A_R, A_2B_R, and A_3_R) and despite the fact that there was some uncertainty about how they were distributed in the cells of the paradigmatic NMJ (Lynge and Hellsten, 2000; Baxter et al., 2005), some of them have been localized in the mouse synapse with confocal immunohistochemistry (Garcia et al., 2005, 2013, 2014; Wright et al., 2009). The confidence of protein localization by immunohistochemistry lies in the specificity of the antibodies used. Though the lack of signal in knockout animals was not investigated, we made an effort to characterize the commercially available antibodies by Western blotting. Our results have shown that the A_1_R receptor is more abundant in adult animals, whereas the A_2A_R receptor predominates in the newborn ones (Garcia et al., 2013). Moreover, the A_2B_R and A_3_R receptors are more expressed in the adult muscles than in the younger ones (Tomàs et al., 2014).

**FIGURE 1:**
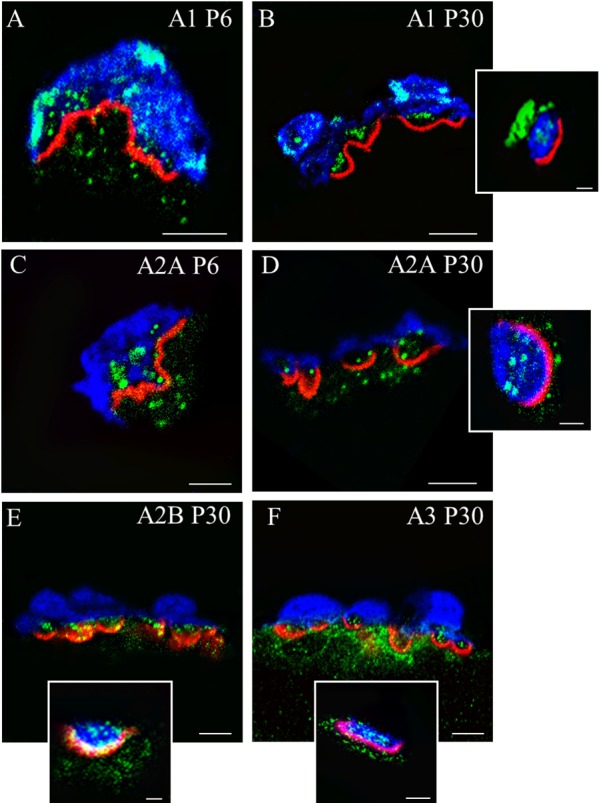
Immunolocalization of A_1_R, A_2A_R, A_2B_R, and A_3_R receptors in the motor nerve terminals on neuromuscular synapses. Immunofluorescence staining and confocal microscopy analysis in newborn (P6) and mature (P30) mice. Triple labeling of the corresponding receptor proteins (green fluorescence) with S-100 (blue fluorescence) and rhodamine-alpha-bungarotoxin (red fluorescence). In the insets (scale bar, 1 μm), Syntaxin labeling (in blue) is showed instead of S-100. **(A–D)** Are single confocal planes from NMJs obtained using plastic embedded semithin cross-sections (0.5–0.7 μm). **(E,F)** Are single confocal planes from a projection image. In the cross-sections of the synaptic boutons, all receptors are present as a fine granular labeling in the space of the nerve terminals between the blue Schwann cell and the red postsynaptic line. In all cases, (see the insets showed as examples), the receptor proteins colocalize with Syntaxin. The bar indicates 10 μm. The images are illustrative examples of the original studies published in Garcia et al. (2014) (Garcia N., Priego M., Hurtado E., Obis T., Santafe M. M., Tomàs M., Lanuza M. A., Tomàs J. Adenosine A2B and A3 receptor location at the mouse neuromuscular junction. *J. Anat*. 225:109–117) and Garcia et al. (2013) (Garcia N., Priego M., Obis T., Santafe M. M., Tomàs M., Besalduch N., Lanuza M. A., Tomàs J. Adenosine A1 and A2A receptor-mediated modulation of acetylcholine release in the mice neuromuscular junction. *Eur. J. Neurosci*. 38:2229–2241).

Immunofluorescence staining coupled with confocal microscopy analysis was performed to determine the localization of A_1_R, A_2A_R (whose function has been further investigated) in P6 and P30 NMJ (**Figure 1**). Moreover, localization of A_2B_R, and A_3_R has been determined in P30 NMJ. By triple labeling of NMJ we stained each one of these protein receptors in green fluorescence together with Syntaxin or S-100 (blue fluorescence) and nAChR (red fluorescence) and we saw that the molecules were present. However, since the components of the NMJ (the nerve terminal, the Schwann cell and the muscle fiber) are juxtaposed, it is not always easy to locate the proteins with precision. Thus, in some cases, to better determine A_1_R and A_2A_R localization, we used plastic embedded semithin cross-sections as a tool for high-resolution together with the triple-labeling immunofluorescence analysis. Briefly, after conventional immunohistochemistry the samples were dehydrated with increasing concentrations of ethanol and acetone, the tissue fragments were embedded in Spurr’s resin in transverse orientation and sections (0.5–0.7 μm thick) were obtained (Lanuza et al., 2007; Garcia et al., 2010c; Besalduch et al., 2013). Images (A–D) correspond to a single image made in a confocal microscope from a 0,5 μm muscle semithin cross section. Images (E–F) show localization of A2BR and A3R using single confocal Z planes from a projection image of at least 10 confocal Z planes obtained every 0,5 μm. As shown in **Figure 1**, these receptors are differentially located in the three cells that configure the NMJs (Garcia et al., 2013, 2014). To the purpose of the present review, it can be noted that the four subtypes of AR are present in the motor endings, which is understood to be a requisite to modulate presynaptic function. However, some AR are localized also in the other synaptic components (for instance A_3_R in the postsynaptic site) and the interpretation of the results here is not a full representation of the AR physiology.

## Adenosine Receptors Role During Development

During the nervous system development there is an overwhelming production of synapses (that may promote connectiviy), followed by an activity-dependent reduction of them. Hebbian competition between axons refines connectivity and increases specificity (Purves and Lichtman, 1980; Jansen and Fladby, 1990; Sanes and Lichtman, 1999; Chen and Regehr, 2000; Nadal et al., 2016a). In newborn animals, skeletal muscle cells are innervated by various motor axons (Ribchester and Barry, 1994) but when the competition ends, the NMJs retain only one axon (Liu et al., 1994; Nguyen and Lichtman, 1996; Chang and Balice-Gordon, 1997; Sanes and Lichtman, 1999; Herrera and Zeng, 2003; Nelson et al., 2003; Wyatt and Balice-Gordon, 2003; Buffelli et al., 2004).

The postsynaptic cell and the terminal Schwann cells may be intermediary in axonal competition. A decrement in polyneuronal innervation occurs at a time when relatively little loss of the nAChR postsynaptic receptors was observed (Lanuza et al., 2002). However, in some cases, local receptor loss has been observed before the corresponding axon loss (Balice-Gordon and Lichtman, 1993). This suggest that pre- and postsynaptic changes are coordinated. Non-myelinating terminal Schwann cells at the NMJ play a role in synapse elimination (reviewed by Lee et al., 2017). A model has been proposed in which the activity of the Schwann cells promote synapse elimination by creating vacant synaptic sites that can be reoccupied by the competing axon terminals.

At the time this process occurs, several signaling mechanisms coordinate the pre- and postsynaptic cell function. First of all, presynaptic mAChR receptors allow direct competitive interaction between nerve endings because of their different activity-dependent ACh secretion (Santafé et al., 2004, 2007, 2009; Nadal et al., 2016a,b; Tomàs J. et al., 2017). Moreover, we also investigated the involvement of presynaptic AR (A_1_R and A_2A_R), which monitor both nerve- and muscle-derived adenosine release during the complex period of axonal elimination around P5-P9 (Garcia et al., 2013; Nadal et al., 2016a,b).

**FIGURE 2:**
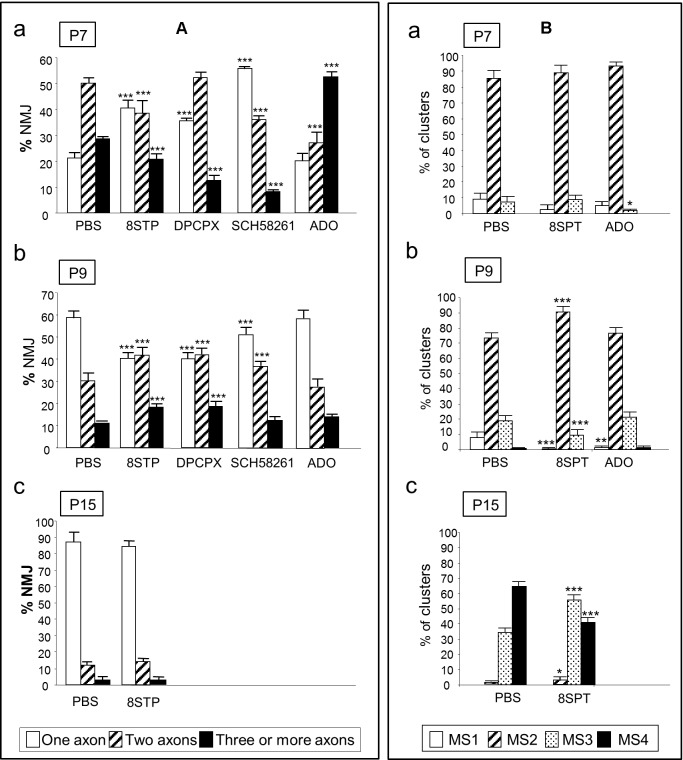
Involvement of AR in axonal elimination and in the morphological maturation of the postsynaptic apparatus. The **(A)** shows the percentage of the singly-, dually-, and triply- (or more) innervated NMJs in the YFP control mice exposed to PBS, and after 2 (P7 in **a**), 4 (P9, in **b**) and in some cases 10 (P15, in **c**) applications (one application every day after P5) of the AR pan-inhibitor 8SPT and the AR agonist adenosine (ADO). We also studied axonal elimination after selectively blocking A_1_R with the antagonist DPCPX and inhibiting A_2A_R with SCH-58261. The **(B)** shows the percentage of the MS1–MS4 maturation stages in the NMJ of the untreated YFP control mice (exposed to PBS), and after 2 (P7 in **a**), 4 (P9, in **b**), and in some cases 10 (P15, in **c**) applications of the 8SPT and ADO. Fisher’s test: ^∗^*p* < 0.05, ^∗∗^*p* < 0.01, ^∗∗∗^*p* < 0.005. This Figure has been adapted and redraw from the Figures 4, 8 in the original article “[Presynaptic muscarinic acetylcholine autoreceptors (M_1_, M_2_, and M_4_ subtypes), adenosine receptors (A_1_ and A_2A_) and tropomyosin-related kinase B receptor (TrkB) modulate the developmental synapse elimination process at the neuromuscular junction]” by [Nadal, L., N. Garcia, E. Hurtado, A. Simó, M. Tomàs, M. A. Lanuza, M. Santafé, and J. Tomàs].” *Mol. Brain.* 2016, 9: 67 (doi: 10.1186/s13041-016-0248-9). The original article is an open access article distributed under the terms of the Creative Commons Attribution License (http://creativecommons.org/licenses/by/2.0), which permits unrestricted use, distribution, and reproduction in any medium, provided the original work is properly cited.

**FIGURE 3:**
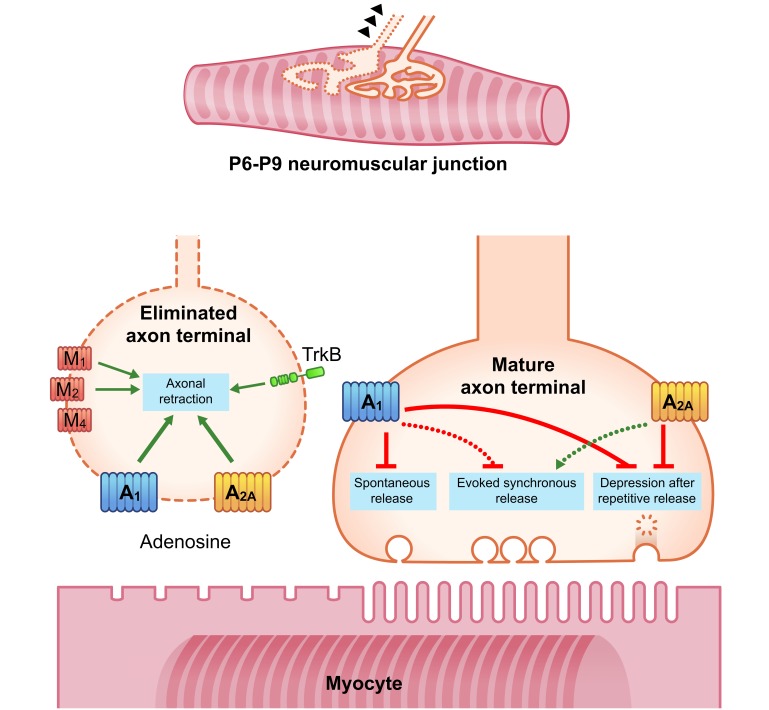
Diagrams showing an overall representation of the data. Between P6-P9 several nerve endings are eliminated and retract whereas one nerve terminal is stabilized. Green arrows indicate stimulation or promotion; red lines indicate inhibition. During the 1st week postnatal an A_1_R- and A_2A_R-mediated tonic acceleration of axonal retraction is observed (green arrows in the eliminated axon terminal). In addition to the role of AR, at least mAChR M_1_-, M_2_-, and M_4_-subtypes and the TrkB receptor are also involved. The downstream pathways integrate the signals related with the competitive interactions and accelerate axonal loss. In the adult NMJ, most experiments on transmitter release have been done in recording conditions that interfere with the synapse function to prevent muscle contraction. In these conditions A_1_R reduces it whereas A_2A_R increases it. Discontinuous lines in the mature axon terminal indicate these changes in conditions of safety factor reduction. In experiments with μ-CgTx-GIIIB only the voltage-dependent sodium channel of the muscle cells was shut-down, thus resulting in non-contractile muscles which have well preserved NMJ physiology and safety factor. In these conditions AR are not coupled to any immediate modulation of evoked neurotransmission. However, AR can restrict spontaneous quantal leak of ACh (A_1_R) and protect synaptic function by reducing the magnitude of depression during repetitive activity (an A_1_R- and A_2A_R-mediated effect).

We evaluated the average number of axonal connections per NMJ from B6.Cg-Tg (Thy1-YFP) (from now YFP) and C57BL/J6 mice. To the flat and accessible mouse LAL muscle, we subcutaneously applied the unselective AR inhibitor 8-(p-sulfophenyl) theophylline (8SPT) and the agonist adenosine between P5-P15 (**Figure 2**). At P7, after the inhibition with 8SPT of the AR, we observed an acceleration of the axonal elimination on the NMJ indicating that, at this point of development, the role of AR is to delay axonal loss (Nadal et al., 2016a). In accordance, exposure to the physiological agonist adenosine resulted in a retardation of axonal elimination (i.e., a significant high number of triple innervated NMJ and a reduction in the number of dual innervated NMJ, **Figure 2Aa**). We also analyzed axonal loss after selectively blocking A_1_R with 8-cyclopentyl-1,3-dipropylxanthine (DPCPX) or A_2A_R with 2-(2-furanyl)-7-(2-phenylethyl)-7H-pyrazolo[4,3-e] [1, 2, 4] triazolo[1,5-c]pyrimidin-5-amine (SCH-58261) (Nadal et al., 2016a,b). Results showed that axonal loss is accelerated by both inhibitors indicating that, in normal conditions, both receptors A_1_R and A_2A_R are related with delaying axonal elimination. However, at P9 (**Figure 2Ab**), the purinergic function accelerates axonal loss to the maximum rate. Therefore, an initial delay in axonal loss at P7 (an A_1_R- and A_2A_R-mediated effect which can be reinforced by exogenously added adenosine) is followed by an A_1_R- and A_2A_R-mediated tonic acceleration of axonal loss at P9 (green arrows in **Figure 3** left, Nadal et al., 2016a). We also investigated the effect of long-term AR perturbation with 8SPT over axon number at P15. Despite the continued presence of the inhibitor, monoinnervation is achieved in about 90% of NMJ at P15 (**Figure 2Ac**) suggesting that axonal competition and loss are differentially modulated (Nadal et al., 2016a).

Axonal elimination is accompanied by changes in the structure of the nicotinic ACh receptor (nAChR) clusters in the postsynaptic site (**Figure 2B**). Based on criteria from previous studies on developing mammalian NMJ (Steinbach, 1981; Slater, 1982a,b; Bewick et al., 1996; Lanuza et al., 2002; Garcia et al., 2011), we defined several maturation stages (MS1–MS4). Changes in nAChR distribution transform the uniform nAChR oval cluster at birth (MS1) into an elongated plaque with some heterogeneities in the density of receptors (MS2). Later, they become into clusters with small zones of low receptor density (MS3) that are not innervated that lead to a mature pattern of independent primary gutters (MS4) (Nadal et al., 2016a). In relation with these changes, we found that the antagonist 8SPT, applied in the period P5–P8, had no effect on the clusters morphology when observed at P7 (**Figure 2Ba**). However, at P9 we found that MS2clusters were increased while the MS1 and MS3 ones decreased (**Figure 2Bb**), which indicates a delay in the transition from MS2 to MS3. At P15 (**Figure 2Bc**) the postsynaptic maturation is partially retained at the MS3 stage. Thus, AR are able to accelerate maturation during the P7–P15 period. Interestingly, the agonist adenosine does not unambiguously change the cluster maturation indicating that the tonic effect of the AR evidenced by using 8SPT is close to their maximum (Nadal et al., 2016a).

In summary, AR are involved in the control of the competitive interactions between nerve endings, possibly helping to determine the winner or the losers but, thereafter, axon loss seems to occur with autonomy.

## Adenosine Receptors Role in the Adult Neurotransmission

Once a NMJ becomes mature and monoinnervated, AR continue modulating neurotransmission. By measuring the activity-dependent efflux of radiolabelled ACh incorporated in nerve endings, Correia-de-Sá et al. (1991) showed that AR control their nerve-stimulated release. Micromolar adenosine levels reduced evoked and/or spontaneous ACh release in frog NMJs (Searl and Silinsky, 2005; Shakirzyanova et al., 2006; Adámek et al., 2010) and also in rat NMJs (Silinsky et al., 1989; De Lorenzo et al., 2006; Pousinha et al., 2010). However, in rat NMJs, submicromolar adenosine concentrations has the opposite effect (Pousinha et al., 2010). Moreover, in other studies done in mice, only very high doses of adenosine (in the range of millimolar) affected neurotransmission (Silinsky, 2004) and, in concordance, some mammalian endplates were insensitive to adenosine (Ginsborg and Hirst, 1972). Therefore, it remains unclear how and when adenosine and AR modulate neurotransmission. The majority of experiments have been done in electrophysiological recording conditions that interfere with the normal function of the NMJ to prevent muscle contraction. For example, in the ACh efflux experiments hemicholinium-3 prevents choline reuptake, high Mg^2+^ concentrations reduce ACh release and d-tubocurarine reduces postsynaptic response. In these conditions, selective agonists and antagonists of the A_1_R and A_2A_R modify ACh release. On the one hand, the A_1_R agonist 2-Chloro-N^6^-cyclopentyladenosine, 1 μM (CCPA) reduces it (Veggetti et al., 2008) whereas the A_2A_R agonist 2-p-(2-Carboxyethyl)- phenethylamino-5′-N-ethylcarboxamidoadenosine hydrochloride hydrate, 1 μM (CGS-21680) increases it (Pousinha et al., 2010). However, A_1_R reduces release (in high Mg^2+^ and curare blockade) when the NMJ is already weakened and because of that, the meaning of this regulation seems unclear (Garcia et al., 2013).

Because of these uncertainties, we induced muscle paralysis with μ-CgTx-GIIIB (Garcia et al., 2013; Tomàs et al., 2014; Obis et al., 2015; Santafé et al., 2015; Hurtado et al., 2017a,b), a specific inhibitor of the sodium channel of the muscle cells which preserves NMJ function (Garcia et al., 2013) and its safety factor. This experimental condition mimics the physiological conditions of this synapse in the living animals except for the absence of the contraction-dependent retrograde influence of the muscle cells (Besalduch et al., 2011; Hurtado et al., 2017a,b). We observed that 25 μM adenosine reduced (50%) the quantal content of ACh release in agreement with other authors (Ginsborg and Hirst, 1972; Ribeiro and Walker, 1975). However, in the nearly normal basal conditions (only test stimulations of 70 pulses at 0.5 Hz every 5 min in the presence of μ-CgTx-GIIIB), none of the purinergic agonists or antagonists had any effect on the evoked ACh release. In particular, we tested non-selective AR agonists and antagonists (adenosine and 8SPT, respectively), A_1_R-selective agonists and antagonists (CCPA 1 μM and DPCPX 100 nM, respectively) and A_2A_R-selective agonists and antagonists (CGS-21680 1 μM and SCH-58261 50 nM, respectively) (Garcia et al., 2013; Tomàs et al., 2014). However, we detected that AR were still functional in reducing the spontaneous release because miniature endplate potentials (MEPP) frequency was increased by SPT8 blockade and decreased by adenosine stimulation, with A_1_Rs playing the main role because only DPCPX increased MEPP frequency (Garcia et al., 2013). In addition, imposed synaptic activity (40Hz for 2 min of supramaximal stimuli) resulted in synaptic depression, an effect reduced by micromolar adenosine but potentiated by blocking AR with 8SPT. Depression in control muscles represents a ∼50% reduction of the endplate potentials (EPP) amplitude and 10 μM adenosine reduces it to a half (Garcia et al., 2013). Surprisingly, we found that depression was not affected by any selective agent, which suggested that both A_1_R and A_2A_R need to collaborate (Garcia et al., 2013; Tomàs et al., 2014). In perspective, the fact that adenosine and 8SPT modified synaptic depression, whereas A_1_R and A_2A_R ligands did not, suggested us that A_2B_R and/or A_3_R could be implicated. Thus, we investigated the A_2B_R-selective antagonist MRS1706 (N-(4-Acetylphenyl)-2-[4-(2,3,6,7-tetrahydro-2,6-dioxo-1,3-dipropyl-1H-purin-8-yl)phenoxy]acetamide, 100 nM) and the A_3_R-selective antagonist MRS1334 (1,4-Dihydro-2-methyl-6-phenyl-4-(phenylethynyl)-3,5-pyridinedicarboxylic acid 3-ethyl-5-[(3nitrophenyl)methyl] ester, 100 nM). We unexpectedly found that A_2B_R and A_3_R neither had any effect on depression (just as the A_1_R and A_2A_R selective antagonists). Thus, we concluded that two or more AR are necessary to protect against depression (Garcia et al., 2013; Tomàs et al., 2014).

In summary, these findings confirmed that in basal conditions AR are not coupled to any immediate modulation of evoked neurotransmission. However, these receptors are still crucial to preserve resources by avoiding the leak of spontaneous quantal ACh, an action which is probably implicated in their protective role against synaptic depression after repetitive activity (Tomàs et al., 2014), (the mature axonal terminal in **Figure 3**).

## Links of Ar With Other Metabotropic Receptors (Development and Adulthood)

Activity-dependent mediators derived from the three cells of the synapse cross the extracellular cleft in all directions to generate signals in target metabotropic receptors. In the NMJ, there are other purinergic receptors apart from AR (Tsim and Barnard, 2002; Todd and Robitaille, 2006), several mAChR (Santafé et al., 2007, 2009; Wright et al., 2009; Garcia et al., 2010b), neurotrophin receptors (Gonzalez et al., 1999; Garcia et al., 2011; Santafé et al., 2015; Nadal et al., 2016a,b) cytokine receptors (Ribchester et al., 1998; Wang et al., 2002; Garcia et al., 2010a, 2012), calcitonin gene-related peptide receptors (Changeux et al., 1992; Lu and Fu, 1995; Gaydukov et al., 2016), glutamate receptors (Thomas and Sigrist, 2012; Personius et al., 2016; Tsentsevitsky et al., 2017) and neuregulin receptors (Loeb, 2003; Kummer et al., 2006; Simeone et al., 2010; Schmidt et al., 2011;Wang et al., 2017). The way a synapse operates is largely the outcome of the confluence of several signaling pathways on intracellular kinases, which phosphorylate protein targets and materialize adaptive changes to modulate transmitter release and the stability of the connection. Therefore, the appropriate knowledge of synaptic behavior needs the integrated albeit complex interpretation of the complementary function of these receptors. Thus, we studied the link and interaction of AR with mAChR and the neurotrophin receptor TrkB in the control of synapse elimination during development and transmitter release in the adult NMJ (Nadal et al., 2016a,b, 2017).

In relation with the synapse elimination process during development, in addition to the role of AR subtypes (A_1_R and A_2A_R) described above, we investigated the involvement of individual mAChR M_1_-, M_2_-, and M_4_-subtypes and the TrkB receptor (Garcia et al., 2010b; Nadal et al., 2016a,b, 2017). Our data indicated that the three receptor sets and all subtypes considered could affect the competition between axon terminals. At P7, for instance, all these receptors taken individually (analyzed through selective inhibition) are involved in favoring initial competition and thus delaying axonal loss. The confluence of the respective downstream pathways can integrate the signals related with the competitive interactions and possibly helps to determine the nerve ending that finally wins and/or the ones that are lost. This competition concludes with the acceleration of the axonal loss 2 days later (around P9; see the eliminated axonal terminal in **Figure 3**).

To study the collaboration of the AR with mAChR and TrkB, we applied two selective antagonists from two different receptors to see the additive or occlusive effects between them at P9 (Nadal et al., 2016a,b, 2017; Tomàs J. et al., 2017; Tomàs J. M. et al., 2017). These experiments showed the existence of a synergistic role between M_1_ and M_4_ mAChR, which potentiates the effect of both AR on axonal elimination. Contrarily, the M_2_ subtype and the TrkB receptor fully occlude the effects of both A_1_R and A_2A_R. Interestingly, when both A_1_R and A_2A_R are blocked at the same time, a mutual occlusion occurs, and the result does not differ from untreated control (Tomàs J. M. et al., 2017).

**FIGURE 4:**
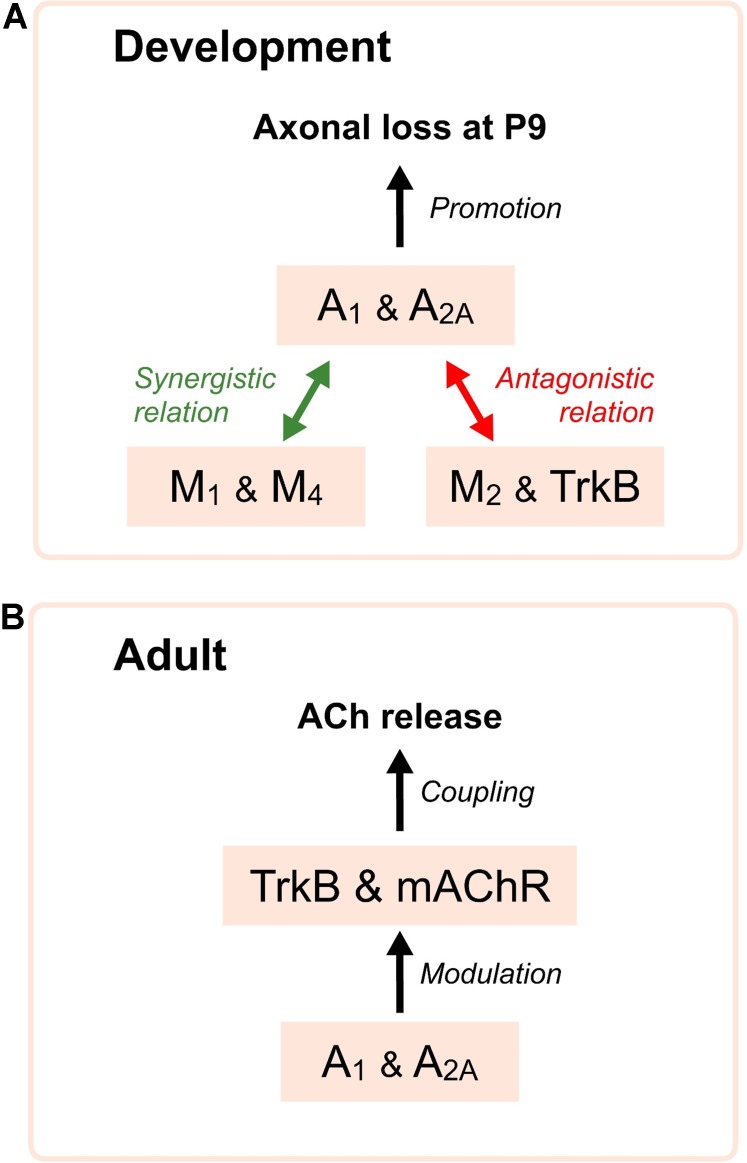
Diagrams showing relations of AR with mAChR and TrkB receptors. In **(A)**, synergistic and antagonistic roles of the AR, mAChR and TrkB receptor during development. AR are necessary to modulate axonal loss and several synergistic (green arrows) and antagonistic (red arrows) relations are clearly observed between all receptors, which affect synapse elimination. In **(B)**, the diagram indicates that the functional integrity of the AR favors the normal operation of the mAChR and the TrkB pathways in the control of the neurotransmitter release in the adulthood.

Thus, both AR are necessary to modulate synapse elimination and several synergistic and antagonistic links are observed between all receptors, which regulate axonal loss (**Figure 4A**). A_1_, M_1_, and TrkB are coupled to stimulate PKC whereas A_2A_, M_2_, and M_4_ inhibit PKA. We hypothesize that a membrane receptor-induced shifting in the protein kinases A and C activity in some nerve endings may play an important role in promoting developmental NMJ maturation.

In the adult NMJ, it is also known that A_1_R and A_2A_R as well as the M_1_-, M_2_-, and M_4_-subtypes of mAChR and the TrkB receptor play several roles in the regulation of transmitter release (Minic et al., 2002; Santafé et al., 2003, 2004, 2007, 2009; Garcia et al., 2010b; Tomàs et al., 2011). By using selective agonists and antagonists we observed that, some receptors (i.e., A_1_R as previously stated) produce minor changes in spontaneous quantal output (see **Figure 3**, right) whereas other receptors (i.e., mAChR) induce major changes in evoked release. Thus, each receptor regulates a given combination of spontaneous, evoked and activity-dependent ACh release. As it has been mentioned before, AR preserve resources by reducing spontaneous leak of neurotransmitter (an A_1_R effect) and normalize the synapse function because stimulation with adenosine reduces the magnitude of depression during repetitive activity. mAChR stabilize the spontaneous quantal output of ACh and also preserve the synapse function by decreasing evoked release (mainly an M_2_ action) and reducing depression. A role for the TrkB receptor is to stabilize the spontaneous quantal leak of ACh but it mainly potentiates evoked release and synaptic potentials (Tomàs et al., 2014).

We also studied the consecutive incubation with two inhibitors affecting two different receptors to see their collaboration in neurotransmission. Adenosine outflow from nerve endings may, through A_1_R, reduce M_2_ effect on ACh release and adenylyl cyclase may be the shared intracellular node between both pathways (Oliveira et al., 2009). We observed that non-specific AR modulation with 8SPT or adenosine abolishes the effect of a second exposure to the unselective mAChR blocker atropine, but also the effect of the M_1_-selective inhibitor pirenzepine and M_2_-selective blocker methoctramine. Thus, the normal operation of the AR is necessary for the normal function of the mAChRs (Tomàs et al., 2014) (**Figure 4B**). However, the same effect is observed in the other way around: a previous blockade of mAChR does not allow applied adenosine to change the above described effect on ACh release in our conditions.

Finally, it has also been reported that adenosine acting through A_2A_ receptors is able to transactivate the TrkB receptor without the need of neurotrophin binding (Lee and Chao, 2001; Lee et al., 2002; Wiese et al., 2007). Thus, AR are also implicated in the neurotrophic TrkB signaling (**Figure 4B**).

## Concluding Remarks

Autoreceptors subtypes are present in the motor nerve endings in the NMJ. During development, AR modulate the competition between axon terminals, helping to define the winner and the losers (Nadal et al., 2016b). To accomplish this function, AR establish several synergistic and antagonistic relations with, at least, the mAChR subtypes and the TrkB receptor which affect synapse elimination. In the mature NMJ, AR help transmitter release by limiting the spontaneous quantal leak of ACh, which mitigates depression during repetitive activity and preserves synaptic function (Tomàs et al., 2014). In addition, the functional integrity of the AR is crucial for the normal operation of the mAChR and the TrkB pathways. These observations underlie the relevance of AR in the NMJ function.

## Ethics Statement

The mice were cared for in accordance with the guidelines of the European Community’s Council Directive of November 24, 1986 (86/609/EEC) for the humane treatment of laboratory animals. All experiments on animals have been reviewed and approved by the Animal Research Committee of the Universitat Rovira i Virgili (Reference number: 0233).

## Author Contributions

LN, EH, AS-O, VC-M, LJ-B, MS, and MT: data collection, quantitative analysis, literature search, data interpretation, and graphic design. NG and ML: statistics. JT, NG, and ML: conception and design, literature search, data interpretation, and manuscript preparation.

## Conflict of Interest Statement

The authors declare that the research was conducted in the absence of any commercial or financial relationships that could be construed as a potential conflict of interest. The reviewer CS and handling Editor declared their shared affiliation.

## Abbreviations

ACh: acetylcholine
AR: adenosine autoreceptors
ATP: adenosine triphosphate
EPP: evoked endplate potentials
LAL: Levator auris longus muscle
mAChR: muscarinic acetylcholine receptor
M_1_: M_1_-type muscarinic acetylcholine receptor
M_2_: M_2_-type muscarinic acetylcholine receptor
M_4_: M_4_-type muscarinic acetylcholine receptor
NMJ: neuromuscular junction
TrkB: tropomyosin-related kinase B receptor
TrkB-Fc: inhibitor recombinant human TrkB-Fc Chimera
